# Comparison of Metabolic Syndrome (MetS) Risk and Nutritional Status According to Menopause Age and the Impact of Socioeconomic Status on MetS Prevalence in Postmenopausal Women: A Cross-Sectional Study Based on the 8th Korea National Health and Nutrition Examination Survey (KNHANES)

**DOI:** 10.3390/nu16070967

**Published:** 2024-03-27

**Authors:** Anna Han, Yean-Jung Choi

**Affiliations:** 1 Department of Food Science and Human Nutrition, Jeonbuk National University, Jeonju 54896, Republic of Korea; annahan8659@jbnu.ac.kr; 2 K-Food Research Center, Jeonbuk National University, Jeonju 54896, Republic of Korea; 3 Department of Food and Nutrition, Sahmyook University, Seoul 01795, Republic of Korea

**Keywords:** socioeconomic, metabolic syndrome, nutrient intake, menopause age, KNHANES

## Abstract

The increased life expectancy and the occurrence of premature menopause prolong the mean postmenopausal phase in women’s lifespans. Although the roles of poor socioeconomic status (SES), anthropometric characteristics, and nutritional status in premature menopause and the health of postmenopausal women are well understood, the differences in nutritional status and metabolic syndrome (MetS) prevalence in postmenopausal women depending on their menopause age are less explored. Furthermore, the association between SES and MetS risk in postmenopausal women is not studied. Thus, this study aimed to compare distinct nutritional status and MetS risk between women with premature menopause and natural menopause. Additionally, the association among SES, health-related lifestyle behaviors (HLBs), and MetS risk in postmenopausal women was studied. This study included 31,799 postmenopausal women from the 8th National Health and Nutrition Examination Survey (KNHANES). The relationship between disease prevalence and nutrient intake of the subjects was analyzed using analysis of variance (GLM), and Scheffé test was performed. Multiple logistic regression analysis was used to evaluate the association among SES, HLBs, and MetS as well as premature menopause. Women with premature menopause showed poor SES, anthropometric characteristics, and HLBs compared with women with natural menopause. Additionally, premature menopausal women had markedly lower intakes of protein, polyunsaturated fatty acid, *n*-3 fatty acid, and β-carotene, but higher intakes of energy, carbohydrate, saturated fatty acid, and sugar than women with natural menopause (*p* < 0.0001). Premature menopausal women showed significantly higher MetS prevalence by having hypertriglyceridemia (*p* < 0.0001), hypertension (*p* = 0.0145), and reduced HDL cholesterol levels (*p* < 0.0001) relative to natural menopausal women. Furthermore, our findings indicate a substantial link among SES, HLBs, and the risk of premature menopause. In postmenopausal women, deteriorating SES and HLBs appear to influence the prevalence of MetS. Notably, our study reveals that higher intakes of protein, calcium, phosphate, and iron are correlated with a lower risk of developing MetS. These observations suggest that proactive nutritional education for premature menopausal women is necessary to improve MetS risk and their nutritional status. Also, SES-dependent interventions regarding nutrition and HLBs in postmenopausal women will be significant to lower MetS risk, MetS-derived chronic disease, and mortality in postmenopausal women.

## 1. Introduction

Menopause is the permanent cessation of menstruation caused by the deprivation of ovarian follicular capability and hormone production (e.g., estrogen and progesterone), resulting in the end of reproductive ability in women [1]. Generally, menopause occurs in middle-aged women (≥50 years) and triggers several physical and emotional changes, including sweating, sleeping issues, and depression [2]. Importantly, women’s life expectancy and the number of cases of premature menopause (<40 years) have been increasing [3,4], lengthening the average postmenopausal period in women’s lifespans. For example, in Korean women, the mean age of menopause was 49.9 years, and the life expectancy was 86.6 years in 2021, which was 83.6 years in 2010 [5]. Moreover, one out of hundreds of Korean women experience premature menopause [6].

Socioeconomic status (SES) is one of the critical factors determining menopausal age [7,8]. In fact, a lower SES was significantly associated with an elevated incidence of premature ovarian insufficiency and early menopause (between ages 40 and 45 years) [8]. The significant roles of SES in menopausal age come from its effects on an individual’s health-related lifestyle behaviors (HLBs, e.g., smoking, physical activities, and alcohol intake), nutritional status, and anthropometric parameters [9,10,11]. For instance, appropriate body weight and body fat percentage are pivotal to maintaining a regular ovulatory cycle and lowering the risk of amenorrhea and premature menopause [12,13]. Additionally, the association between smoking and premature menopause has been consistently reported [14].

Due to the abrupt end of hormone production, menopause leads to metabolic dysregulation, such as hyperlipidemia and insulin resistance, increasing the risk of multiple chronic diseases (cardiovascular disease and diabetes) in postmenopausal women [15,16]. Indeed, about 22% of postmenopausal women show metabolic syndrome (MetS) by having higher fasting glucose level (≥100 mg/dL) and systolic blood pressure (≥130 mmHg) [16]. Therefore, the importance of the nutritional condition of postmenopausal women has been emphasized to maintain health and prevent the incidence of chronic disease. For instance, adequate consumption of antioxidant nutrients (e.g., vitamin A, and vitamin C) is positively correlated with bone health in postmenopausal women [17,18]. Although the association between the nutritional condition and diverse menopause-related health issues in postmenopausal women has been well explored, differences in the nutritional status and MetS prevalence of postmenopausal women depending on their menopausal age are less investigated. Moreover, according to Choi and colleagues, poor SES markedly elevates the osteoporosis risk in postmenopausal women [19]; however, the impact of SES on the MetS risk in postmenopausal women is unexplored.

Therefore, utilizing recent nationally representative data (the 8th Korea National Health and Nutrition Examination Survey (KNHANES VIII-2), 2020), the current study aimed to (1) compare the differences in socioeconomic, HLBs, and anthropometric parameters between women with premature and natural menopause; (2) compare differences in average nutrient intake and MetS risk between women with premature and natural menopause; and (3) scrutinize the association among SES, HLBs, and MetS risk in postmenopausal women.

## 2. Materials and Methods

### 2.1. Study Design and Participants

This study used raw data from the 8th KNHANES (2020). The KNHANES is an extensive, cross-sectional survey targeting individuals aged 1 year and older. Conducted by the Korea Centers for Disease Control and Prevention (KCDC) since 1998, this nationwide study aims to uncover health-related factors through a combination of health examinations, interviews, and nutritional assessments. Each generation of the survey encompasses approximately 2000 to 3000 South Korean participants.

**Figure 1:**
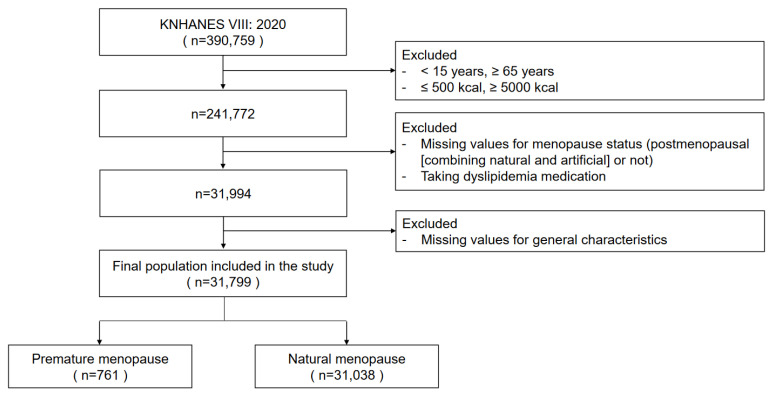
Flowchart depicting the selection process of study participants.

Among the total subjects, 164,088 postmenopausal women who did not menstruate were extracted after excluding those women aged 15 to 65 who were surveyed by the Korean Dietary Reference Intake Index (KDRIs) who answered before menarche, without menstruation, or with an unknown menstrual status. Among them, 31,994 people with natural menopause and artificial menopause were selected for the amenorrhea question. Artificial menopause refers to cases of artificial menopause due to female diseases. In this study, cases of menopause due to artificial causes, such as oophorectomy and hysterectomy, were included. Among them, subjects taking drugs that may affect lipid metabolism were excluded. The final study subjects were classified into 761 women with early menopause and 31,038 women with normal menopause based on the age of 40 at the time of menopause (Figure 1).

### 2.2. Research Contents and Methods

For the general information of the research subjects, income level, education level, and marital status investigated in the KNHANES were used for analysis. For lifestyle, data on drinking experience and frequency, smoking status, and physical activity level were used for analysis.

As for physical characteristics, height, weight, waist circumference (WC), and body mass index (BMI), which were investigated through a physical examination survey, were used. In addition, data investigating the subject’s nutrient intake and prevalence of MetS components were used for analysis. A BMI of less than 25 was classified as normal, whereas a BMI of 25 or more was classified as obese. Additionally, a fasting blood glucose of less than 100 mg/dL was classified as normal, and a fasting blood sugar value higher than 100 mg/dL (≥100 mg/dL) with a doctor’s diagnosis or taking hypoglycemic drugs or insulin injections was classified as diabetic condition. Hypercholesterolemia (HyperCHL) is noted when total cholesterol is 240 mg/dL or higher or cholesterol-lowering drugs are taken. Hypertriglyceridemia (HyperTG) is noted when triglyceride is 200 mg/dL or higher. Hypertension is noted with a blood pressure of 130/85 mmHg or higher. Low high-density lipoprotein cholesterol (HDL-CHL) hyperemia was defined as less than 50 mg/dL.

MetS was defined based on the modified NCEP ATP III following Asian standards for abdominal obesity [20] and metabolic syndrome and noted when more than three of the five criteria were met: (1) elevated blood pressure (average systolic blood pressure > 130 mmHg or diastolic blood pressure > 85 mmHg); (2) low serum concentrations of high-density lipoprotein (HDL) cholesterol (<50 mg/dL for women); (3) serum triglyceride (TG) level (≥150 mg/dL); (4) fasting blood glucose concentrations (≥100 mg/dL); (5) abdominal obesity (waist circumferences for women of ≥85 cm).

The 24-h recall survey was carried out via in-person interviews by trained professionals. To improve recall abilities and gather detailed data on survey items, supplementary materials were utilized during the survey process. A total of 26 nutrients, including energy, protein, fat, and carbohydrates, from the food intake survey using the 24-h recall method of KNHANES were included in the nutrient intake survey.

### 2.3. Data Analysis

Statistical processing of all data was performed using SAS (release 9.4; SAS Institute, Cary, NC, USA) 9.4 program. Each variable was analyzed using the composite sample design data analysis method considering the colony variables, stratification variables, and weights presented in the 8th KNHANES. Descriptive statistics were expressed as mean, frequency, t-test, and standard deviation. Descriptive statistics were presented for height, weight, WC, and BMI, and the chi-square test was performed by classifying all women as postmenopausal, early menopausal, and normal menopausal. In addition, the prevalence of MetS components according to early menopause and general menopause was compared using a t-test. The relationship between MetS prevalence and the nutrient intake of the subjects was analyzed using analysis of variance (GLM), and Scheffé test was carried out. In our analysis, we employed univariate logistic regression to evaluate the associations among SES, HLBs, MetS, and premature menopause. This assessment was adjusted for age and BMI to account for their potential confounding effects. Additionally, in our forest plot analysis, which focused on the relationship between nutrient intakes and MetS, we made adjustments for age, BMI, and energy intake. These adjustments were crucial to isolate the effects of nutrient intake on MetS while controlling for these significant variables. The statistical significance of all data analysis results was analyzed based on *p* < 0.05.

## 3. Results

### 3.1. Sociodemographic and Anthropometric Characteristics According to Menopausal Status

Table 1 shows the sociodemographic features of the subjects. At the time of the survey, women with premature menopause had an average current age of 56.114 ± 9.850 years, while women experiencing natural menopause had an average current age of 57.276 ± 4.365 years. The average age of menopause among women with premature menopause was 36.223 ± 2.709 years, and the value was 50.622 ± 3.252 years for naturally menopausal women. Based on menopausal status, there were significant differences in age, income level, education, alcohol drinking status, smoking, walking, leisure-related moderate-intensity physical activities, and self-assessment of health (*p* < 0.0001). Women with premature menopause showed the highest proportion at the middle-high income level (51.25%) and ≤elementary school education (40.87%), while naturally menopausal women had the highest distribution at the high-income level (44.78%) and high school education (43.00%). Compared with naturally menopausal women, premature menopausal women showed markedly higher levels of heavy alcohol drinking (premature: 4.86% vs. natural: 2.93%), current smoking (premature: 9.33% vs. natural: 2.19%), not walking more than 5 days a week (premature: 70.17% vs. natural: 55.86%), leisure-related moderate-intensity physical activities (premature: 34.30% vs. natural: 25.16%), and poor self-assessment of health (premature: 32.72% vs. natural: 17.85%). Additionally, women with premature menopause had lower values in the good (premature: 17.08% vs. natural: 27.74%) self-assessment of health than women with natural menopause.

Participants’ anthropometric characteristics based on menopausal status are shown in Table 2. Compared to naturally menopausal women, premature menopausal women had significantly lower weight (*p* = 0.0238), WC (*p* < 0.0001), and BMI (*p* = 0.0003).

### 3.2. Prevalence of Metabolic Syndrome Components Based on Menopausal Status

Details on the prevalence of MetS components of the subjects according to menopausal status are shown in Table 3. Women with natural menopause had a markedly higher prevalence of obesity (*p* < 0.0001), diabetes (*p* < 0.0001), and HyperCHL (*p* < 0.0001), also higher HDL cholesterol (*p* < 0.0001) than women with premature menopause. On the other hand, the prevalence of HyperTG (*p* < 0.0001) and hypertension (*p* = 0.0145) was higher in premature menopausal women compared to naturally menopausal women. Women with natural menopause showed significantly lower levels of metabolic diagnosis compared to women with premature menopause (>3, premature: 19.97% vs. natural: 9.62%).

### 3.3. Average Daily Nutrient Intake According to Menopausal Status

There were significant differences in daily nutrient intake based on menopausal status (Table 4). Premature menopausal women had significantly higher average daily intakes of energy (*p* < 0.0001), carbohydrate (*p* < 0.0001), saturated fatty acid (SFA, *p* < 0.0001), fiber (*p* < 0.0001), sugar (*p* < 0.0001), calcium (*p* = 0.0014), phosphorus (*p* < 0.0001), retinol (*p* = 0.0297), and thiamin (*p* < 0.0001) than women with natural menopausal women. Contrastingly, naturally menopausal women showed markedly higher mean daily intakes of water (*p* = 0.0258), protein (*p* < 0.0001), polyunsaturated fatty acid (PUSFA, *p* = 0.0004), *n*-3 fatty acid (*p* < 0.0001), iron (*p* < 0.0001), sodium (*p* < 0.0001), vitamin A (*p* < 0.0001), β-carotene (*p* < 0.0001), and riboflavin (*p* < 0.0001) compared with premature menopausal women.

### 3.4. Factors Associated with the Risk of Premature Menopause

Table 5 shows the association among SES, HLBs, and premature menopause risk. The prevalence of premature menopause was significantly 0.209 times lower in middle-high income level (95% CI = 0.122–0.359) and 0.009 times lower in high-income level (95% CI = 0.003–0.023) compared to the reference group (lower-middle). Education level was also significantly associated with premature menopause risk. Compared to the reference group (≤middle school), the prevalence of premature menopause was markedly 2.454 times higher in ≥ high school (95% CI = 1.726–3.489). Additionally, current smoking status was noticeably related to premature menopause prevalence (OR = 4.230; 95% CI = 3.175–5.636). Furthermore, compared to the reference group (<5 days/week), premature menopause risk was 0.675 times lower in the group that walked more than 5 days/week.

### 3.5. Factors Associated with the Risk of Metabolic Syndrome in Postmenopausal Women

The association among SES, HLBs, and MetS risk is shown in Table 6. The prevalence of MetS in postmenopausal women was significantly associated with income level. The risk of MetS was 1.816 times higher in the middle-high income group (95% CI = 1.417–2.327) and 2.679 times higher in the high-income group (95% CI = 1.816–3.951) compared to the reference group, which was the lower-middle income level. The risk of MetS in postmenopausal women was significant and 0.764 times lower in ≥ high school (95% CI = 0.637–0.915) than the reference group (≤middle school). In addition, heavy alcohol drinking status (OR = 2.684; 95% CI = 2.148–3.353) and current smoking status (OR = 0.168; 95% CI = 0.115–0.245) were also linked with MetS prevalence in postmenopausal women with statistical significance. Also, walking status and leisure-related moderate-intensity physical activities were strongly associated with MetS risk in postmenopausal women. Compared to the reference group (<5 days/week), the prevalence of MetS in postmenopausal women was 0.524 times lower in the group that walked more than 5 days/week (95% CI = 0.479–0.573) with statistical significance. Self-assessment of health also had a statistical association with MetS risk in postmenopausal women.

**Figure 2:**
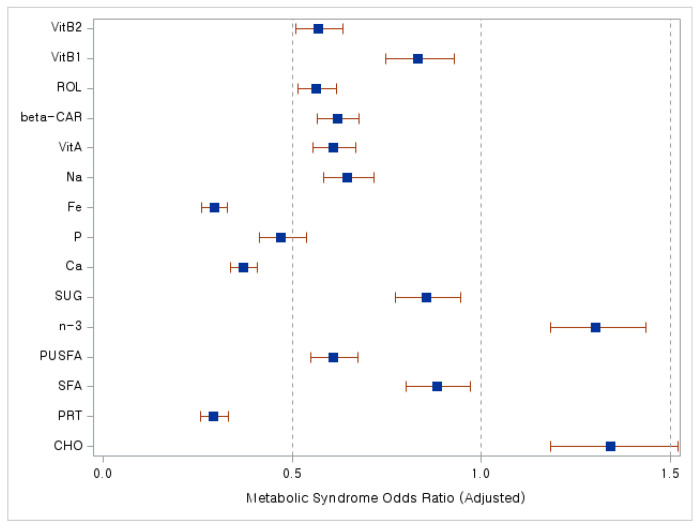
Analysis of metabolic syndrome (MetS) risk in postmenopausal women relative to nutrient intake. A forest plot. Abbreviations: CHO, carbohydrate; PRT, protein; SFA, saturated fatty acids; PUSFA, polyunsaturated fatty acids; *n*-3, omega-3 fatty acids; SUG, sugar; Ca, calcium; P, phosphate; Fe, iron; Na, sodium; VitA, vitamin A; beta-CAR, beta-carotene; ROL, retinol; VitB1, vitamin B1; VitB2, vitamin B2. Blue squares indicate adjusted odds ratio (OR) values, while red lines represent 95% confidence interval (CI) values.

We subsequently performed regression analysis to examine if daily nutrient intake, previously identified as varying based on menopausal status, correlates with the risk of MetS in postmenopausal women. The findings are presented in a forest plot (Figure 2). Observing Figure 2, it is evident that higher intakes of protein, calcium, phosphate, and iron are strongly associated with a reduced risk of MetS.

## 4. Discussion

The current study analyzed the latest nationally representative data (the 8th KNHNES, 2020) and reported the following: (1) the distinct characteristics of SES, HLBs, and anthropometric parameters depending on menopausal age; (2) the differences in MetS prevalence and average nutrient intake relying on menopausal age; (3) the association among SES, HLBs, premature menopause, and the MetS risk in postmenopausal women.

Health inequality has been reported in many countries [21,22]. Individuals having lower SES are likely to have more health issues than those having higher SES due to the worse maintenance in HLBs, nutritional status, and anthropometric characteristics [9,10]. For example, insufficient SES and HLBs are the pivotal factors of premature menopause [7,8]. Consistent with earlier reports, the present study also found that premature menopausal women have markedly poor SES, anthropometric features, and HLBs compared to naturally menopausal women; furthermore, there was a strong association among SES, HLBs, and the risk of premature menopause. As income and education levels increase, the risk of premature menopause was markedly decreased compared to the reference group. Future prospective studies should investigate whether HLB interventions could delay the menopausal age in women having a lower SES as this information is required to investigate the causal relationship of those variables. Furthermore, to improve premature menopause prevalence, it will be critical to inform women regarding the importance of maintenance of proper body weight and body fat percentage especially with young generations showing a high prevalence of severe eating disorders (e.g., anorexia nervosa) [23].

In postmenopausal women, adequate nutrient intake is important to maintain health, ameliorate menopausal-related symptoms, and prevent chronic disease [24,25]. In this study, compared to naturally menopausal women, premature menopausal women showed significantly higher intakes of energy, carbohydrate, SFA, and sugar, but lower consumption of protein, PUSFA, *n*-3 fatty acid, and β-carotene with statistical significance. As a possible outcome of the aforementioned dietary pattern, women with premature menopause had markedly higher metabolic diagnoses by having an elevated risk of HyperTG and hypertension and lower HDL-CHL levels than women with natural menopause. Since premature menopausal women have a longer mean of the menopausal period than naturally menopausal women, nutritional education for premature menopausal women should be actively performed to prevent MetS and lower the chronic disease risk of this population. Indeed, one year of nutritional intervention in overweight/obese postmenopausal women significantly improves body and body fat weight [26]. Moreover, a low-fat diet with increased levels of fruits and grains in postmenopausal women strongly reduced the incidences of cardiovascular disease, diabetes, and breast cancer [24].

Generally, a lower SES is strongly related to negative dietary patterns because it causes less intake of meat, fruits, and vegetables, but more snack consumption [27]; furthermore, there is a significant association between SES and smoking rate [28], subsequently elevating the risk of MetS and chronic disease incidence. Lately, it has been demonstrated that the prevalence of osteoporosis in postmenopausal women was increased with lower SES due to poor knowledge regarding osteoporosis [19]. The present study also observed that worse SES and HLBs noticeably increase the risk of MetS in postmenopausal women. Interestingly, this study found that high-income levels increased MetS prevalence in postmenopausal women. This potentially occurs because individuals with higher SES chronically suffer from psychosocial stress [29], and it has been presented that higher psychosocial stress accelerates bone loss in postmenopausal women [30]. Considering the deleterious outcomes of MetS in postmenopausal women, proactive SES-dependent nutritional and HLB education, including psychosocial stress control, in postmenopausal women will be necessary to improve the prevalence of MetS, MetS-induced chronic disease, and mortality in postmenopausal women.

## 5. Conclusions

Utilizing the 8th KNHANES, this study investigated the differences in socioeconomic, HLBs, and anthropometric indicators between premature and naturally menopausal women. The current study also studied the distinct average nutrient intake and prevalence of MetS between women with premature and natural menopause. Most importantly, this study reported the association between SES and the risk of MetS in postmenopausal women. However, the present study has several limitations. First, the cause of premature menopause was not considered (natural menopause vs. artificial menopause). Furthermore, this study cannot explain causality among the variables as it was a cross-sectional study utilizing the 8th KNHANES. Finally, the survey time and menopause period are not matched, especially for premature menopausal women. In the future, longitudinal and prospective studies are required to understand the causal relationship among the variables and to offer concrete basic information for (1) menopause age-based and (2) SES-dependent dietary guidelines and lifestyle interventions for postmenopausal women.

## Figures and Tables

**Table 1:** Health-associated and socioeconomic characteristics of the participants by menopausal status.

	Premature Menopause (N = 761)	Natural Menopause (N = 31,038)	*p* ^(1)^
Age at current ^(2)^	56.114 ± 9.850	57.276 ± 4.365	<0.0001
Age at menopause	36.223 ± 2.709	50.622 ± 3.252	<0.0001
	N (%)		
Age at current			<0.0001
15 ≤ age < 30	22 (2.89%)	0 (0.00%)	
30 ≤ age < 40	53 (6.96%)	0 (0.00%)	
40 ≤ age < 50	104 (13.67%)	1675 (5.40%)	
50 ≤ age < 65	582 (76.48%)	29,363 (94.60%)	
Age at menopause			<0.0001
age < 50	761 (100.00%)	9761 (31.45%)	
50 ≤ age < 60	0 (0.00%)	21,277 (68.55%)	
Income level			0.0003
Low	23 (3.02%)	3446 (11.10%)	
Low-middle	183 (24.05%)	5385 (17.35%)	
Middle-high	390 (51.25%)	8308 (26.77%)	
High	165 (21.68%)	13,899 (44.78%)	
Education			<0.0001
≤Elementary school	311 (40.87%)	2622 (8.45%)	
Middle school	86 (11.30%)	4453 (14.35%)	
High school	252 (33.11%)	13,345 (43.00%)	
≥College	112 (14.72%)	10,618 (34.21%)	
Heavy alcohol drinking			0.0019
Yes	37 (4.86%)	909 (2.93%)	
No	724 (95.14%)	30,129 (97.07%)	
Current smoking			<0.0001
Yes	71 (9.33%)	679 (2.19%)	
No	690 (90.67%)	30,359 (97.81%)	
Walking			<0.0001
<5 days/week	534 (70.17%)	17,293 (55.86%)	
≥5 days/week	227 (29.83%)	13,664 (44.14%)	
Leisure-related physical activities (moderate-intensity)			<0.0001
Yes	261 (34.30%)	7810 (25.16%)	
No	500 (65.70%)	23,228 (74.84%)	
Self-assessment of health			<0.0001
Good	130 (17.08%)	8609 (27.74%)	
Moderate	382 (50.20%)	16889 (54.41%)	
Poor	249 (32.72%)	5540 (17.85%)	

^(1)^ Different between two groups at α = 0.05 by Mantel–Haenszel chi-square test; ^(2)^ means ± standard deviation.

**Table 2:** Anthropometric characteristics of the participants by menopausal status.

	Premature Menopause (N = 761)	Natural Menopause (N = 31,038)	*p* ^(1)^
Height (cm) ^(2)^	157.493 ± 6.793	157.070 ± 5.644	0.0424
Weight (kg)	57.500 ± 8.087	58.177 ± 8.169	0.0238
Waist circumference (cm)	78.627 ± 6.851	80.999 ± 8.238	<0.0001
Body mass index (kg/m^2^)	23.171 ± 2.967	23.584 ± 3.153	0.0003

^(1)^ Different between two groups at α = 0.05 by ANCOVA test; ^(2)^ means ± standard deviation.

**Table 3:** Prevalence of metabolic syndrome components of the participants by menopausal status.

	Premature Menopause (N = 761)		Natural Menopause (N = 31,038)		
	N (%)				*p* ^(1)^
Obesity					<0.0001
Yes	149 (19.58%)		9564 (30.81%)		
No	612 (80.42%)		21,474 (69.19%)		
HyperTG ^(2)^					<0.0001
Yes	260 (34.17%)		7441 (23.97%)		
No	501 (65.83%)		23,597 (76.03%)		
Diabetes					<0.0001
Yes	198 (26.02%)		11,027 (35.53%)		
No	563 (73.98%)		20,011 (64.47%)		
HyperCHL ^(3)^					<0.0001
Yes	100 (13.14%)		6889 (22.20%)		
No	661 (86.86%)		24,149 (77.80%)		
Hypertension					0.0145
Yes	238 (31.27%)		8466 (27.28%)		
No	523 (68.73%)		22,572 (72.72%)		
HDL-cholesterol					<0.0001
≥50	379 (49.80%)		19,633 (63.25%)		
<50	382 (50.20%)		11,405 (36.75%)		
Metabolic diagnosis					<0.0001
≤3	609 (80.03%)		28,052 (90.38%)		
>3	152 (19.97%)		2986 (9.62%)		

^(1)^ Different between two groups at α = 0.05 by Mantel–Haenszel chi-square test; ^(2)^ HyperTG, hypertriglyceridemia; ^(3)^ HyperCHL, hypercholesterolemia.

**Table 4:** Daily average nutrient intake of the participants by menopausal status.

	Premature Menopause (N = 761)	Natural Menopause (N = 31,038)	*p* ^(1)^
Energy (Kcal) ^(2)^	1874.628 ± 21.788	1738.942 ± 3.413	<0.0001
Water (g)	1113.319 ± 14.807	1146.723 ± 2.318	0.0258
Carbohydrate (g)	281.359 ± 1.687	267.280 ± 0.264	<0.0001
Protein (g)	60.367 ± 0.556	66.557 ± 0.087	<0.0001
Fat (g)	43.600 ± 0.602	44.299 ± 0.094	0.2519
SFA (g) ^(3)^	14.309 ± 0.246	13.247 ± 0.038	<0.0001
MUSFA (g) ^(4)^	13.594 ± 0.244	14.001 ± 0.038	0.0995
PUSFA (g) ^(5)^	11.621 ± 0.194	12.315 ± 0.030	0.0004
n-3 Fatty acid (g)	1.698 ± 0.054	2.150 ± 0.009	<0.0001
n-6 Fatty acid (g)	9.905 ± 0.168	10.139 ± 0.026	0.1673
Cholesterol (mg)	233.549 ± 5.412	242.466 ± 0.847	0.1036
Fiber (g)	34.776 ± 0.365	29.790 ± 0.057	<0.0001
Sugar (g)	81.072 ± 1.093	63.613 ± 0.171	<0.0001
Calcium (mg)	540.839 ± 7.113	517.873 ± 1.114	0.0014
Phosphorus (mg)	1151.231 ± 7.961	1067.583 ± 1.245	<0.0001
Iron (mg)	9.275 ± 0.147	9.958 ± 0.023	<0.0001
Sodium (mg)	2173.107 ± 49.323	3320.482 ± 7.723	<0.0001
Potassium (mg)	3043.785 ± 33.109	3075.978 ± 5.184	0.3368
Vitamin A (μg RAE ^(6)^)	385.004 ± 18.250	477.881 ± 2.857	<0.0001
β-Carotene (μg)	2403.778 ± 118.102	3887.748 ± 18.491	<0.0001
Retinol (μg)	184.655 ± 14.029	153.783 ± 2.196	0.0297
Thiamin (mg)	1.185 ± 0.014	1.053 ± 0.002	<0.0001
Riboflavin (mg)	1.449 ± 0.018	1.552 ± 0.003	<0.0001
Niacin (mg)	11.440 ± 0.141	11.475 ± 0.022	0.8106
Folate (μg DFE ^(7)^)	352.823 ± 4.556	354.893 ± 0.713	0.6536
Vitamin C (mg)	77.730 ± 2.486	80.035 ± 0.389	0.3597
Energy distribution			
%Carbohydrate	63.909 ± 0.356	61.960 ± 0.056	<0.0001
%Protein	13.981 ± 0.130	15.395 ± 0.020	<0.0001
%Fat	22.110 ± 0.296	22.645 ± 0.046	0.0741

^(1)^ Different between two groups at α = 0.05 by ANCOVA test adjusted for age, BMI, and energy (except energy); ^(2)^ Age, BMI, and energy (except energy)-adjusted least squares means (LSmeans); ^(3)^ SFA, saturated fatty acid; ^(4)^ MUSFA, monosaturated fatty acid; ^(5)^ PUSFA, polyunsaturated fatty acid; ^(6)^ RAE, retinol activity equivalent; ^(7)^ DFE, dietary folate equivalent.

**Table 5:** The association between risk factors and premature menopause.

Variables	Premature Menopause (N = 761)		*p*
	Adjusted OR ^(1)^	95% CI	
Body mass index (kg/m^2^)	0.954	0.932–0.978	0.0002
Income level			
Lower-middle	1		
Middle-high	0.209	0.122–0.359	<0.0001
High	0.009	0.003–0.023	<0.0001
Education			
≤Middle school	1		
≥High school	2.454	1.726–3.489	<0.0001
Heavy alcohol drinking			
Yes	1.375	0.953–1.984	0.0885
No	1		
Current smoking			
Yes	4.230	3.175–5.636	<0.0001
No	1		
Walking			
<5 days/week	1		
≥5 days/week	0.675	0.572–0.798	<0.0001
Leisure-related physical activities (moderate-intensity)			
Yes	2.292	1.946–2.698	<0.0001
No	1		
Self-assessment of health			
Good or moderate	1		
Poor	1.175	0.854–1.617	0.3216

^(1)^ Adjusted for age, BMI, income level, education, alcohol drinking status, current smoking status, walking, leisure-related moderate-intensity physical activities, and self-assessment of health.

**Table 6:** The association between risk factors and metabolic syndrome in postmenopausal women.

Variables	Metabolic Syndrome (N = 3138)		*p*
	Adjusted OR ^(1)^	95% CI	
Body mass index (kg/m^2^)	1.398	1.380–1.417	<0.0001
Income level			
Lower-middle	1		
Middle-high	1.816	1.417–2.327	0.0470
High	2.679	1.816–3.951	<0.0001
Education			
≤Middle school	1		
≥High school	0.764	0.637–0.915	0.0034
Heavy alcohol drinking			
Yes	2.684	2.148–3.353	<0.0001
No	1		
Current smoking			
Yes	0.168	0.115–0.245	<0.0001
No	1		
Walking			
<5 days/week	1		
≥5 days/week	0.524	0.479–0.573	<0.0001
Leisure-related physical activities (moderate-intensity)			
Yes	0.525	0.464–0.594	<0.0001
No	1		
Self-assessment of health			
Good or moderate	1		
Poor	1.246	1.050–1.479	0.0120

^(1)^ Adjusted for age, BMI, income level, education, alcohol drinking status, current smoking status, walking, leisure-related moderate-intensity physical activities, and self-assessment of health.

## Data Availability

All data files are available from the Korea Centers for Disease Control and Prevention database through the following URL: https://knhanes.kdca.go.kr/knhanes/sub03/sub03_02_05.do (assessed on 29 January 2024). Any person, including an international researcher who signs up for membership, can obtain raw data from this website. However, the data access process and user manual are only written in Korean.

## Abbreviations

SES, socioeconomic status; HLB, health-related lifestyle behavior; MetS, metabolic syndrome; KNHANES, Korea National Health and Nutrition Examination Survey; KDRIs, Korean Dietary Reference Intake Index; WC, waist circumference; BMI, body mass index; HyperCHL, hypercholesterolemia; HyperTG, hypertriglyceridemia; HDL-CHL, high-density lipoprotein cholesterol; SFA, saturated fatty acid; MUSFA, monosaturated fatty acid; PUSFA, polyunsaturated fatty acid; RAE, retinol activity equivalent; DFE, dietary folate equivalent; and CI, confidence interval.
